# Neuropsychiatric and Cardiac Complications in Post-operative Antibiotic Therapy in Elderly Patients Undergoing Otorhinolaryngological Surgeries: A Review of Frailty Risk Indices

**DOI:** 10.7759/cureus.69765

**Published:** 2024-09-19

**Authors:** Shraddha Jain, Ayushi Ghosh Moulic

**Affiliations:** 1 Otorhinolaryngology, Jawaharlal Nehru Medical College, Datta Meghe Institute of Higher Education and Research, Wardha, IND

**Keywords:** antibiotics, cardiac manifestation, frailty, frailty index, geriatrics, neuropsychiatric manifestation, otorhinolaryngology

## Abstract

With the increase in life expectancy and awareness, more otorhinolaryngologic surgeries are being performed in the elderly population. Otorhinolaryngologic surgeries like septoplasty, tympanoplasty, and dacryocystorhinostomy (DCR) surgeries are elective surgeries for improving the quality of life. However, post-operative medication can entail a risk to life or serious side effects in elderly patients. It is seen that before any surgery, the patients have undergone various tests and investigations to monitor the nephrotoxicity and hepatotoxicity primarily to gauge medication clearance and organ damage. However, the effects of various post-operative medications on the central nervous system and cardiovascular system are less well-discussed. Harmful effects of various drugs especially antibiotics given post-operatively in otorhinolaryngology on the central nervous and cardiovascular system are not frequently reported, and the underlying mechanisms may be unclear or conflicting. Older individuals have a greater chance of experiencing serious drug reactions due to physiologic changes affecting pharmacokinetic processes. Some frailty risk indices can be used to determine the cognitive and physiological conditions in geriatric patients so that the outcome of using antibiotics in the post-operative period on the neuropsychiatric and cardiovascular systems can be predicted. This review aims to summarise the research on the neurotoxic and cardiac effects of antibiotics used in otorhinolaryngology practice in the post-operative period in elderly patients, with a focus on signs of psychosis, delirium, cognitive impairment, syncope, cardiac arrest, angina-like symptoms, etc. This review also studies some frailty risk indices that can be used to predict the neuropsychiatric and cardiac side effects due to polypharmacy, especially antibiotics. Hence, post-operative risks can be predetermined and a protocol for further management can be established.

## Introduction and background

Different types of harm can occur in the elderly as a result of taking various drugs, including nephrotoxicity, cardiac toxicity, and neurotoxicity. In otorhinolaryngology, antibiotics, analgesics, and antihistamines are the most commonly prescribed post-operative drugs. The adverse drug reactions (ADRs) that occur in the geriatric population after surgery are mainly due to changes in pharmacokinetic parameters such as drug distribution, metabolism, excretion, and protein binding [1]. The liver and kidney functioning are equally responsible for drug clearance, and any changes in these organs can lead to toxicity due to drug administration. Neurologic examinations often reveal impairments in motor and sensory functions, while cellular damage is evident through axonopathy and cell death, indicating the severe impact of neurotoxic agents on the nervous system [1].

As a person reaches the age of 75, their body fat can quadruple along with their muscle mass loss, which alters the pharmacokinetics of lipophilic medications leading to changes manifesting as distortions in drug absorption, distribution, metabolism, elimination, and protein binding, all of which significantly elevate the risk of ADRs [1]. As a person ages, various frailty indices have been utilized to diagnose geriatric patients' physical and mental components [2,3]. These indices give an overall assessment of a patient's physical, psychological, and functional condition, which can be used to predict the neuropsychiatric and cardiac manifestations of antibiotics post-otorhinolaryngologic surgeries.

## Review

Search strategy

**Figure 1:**
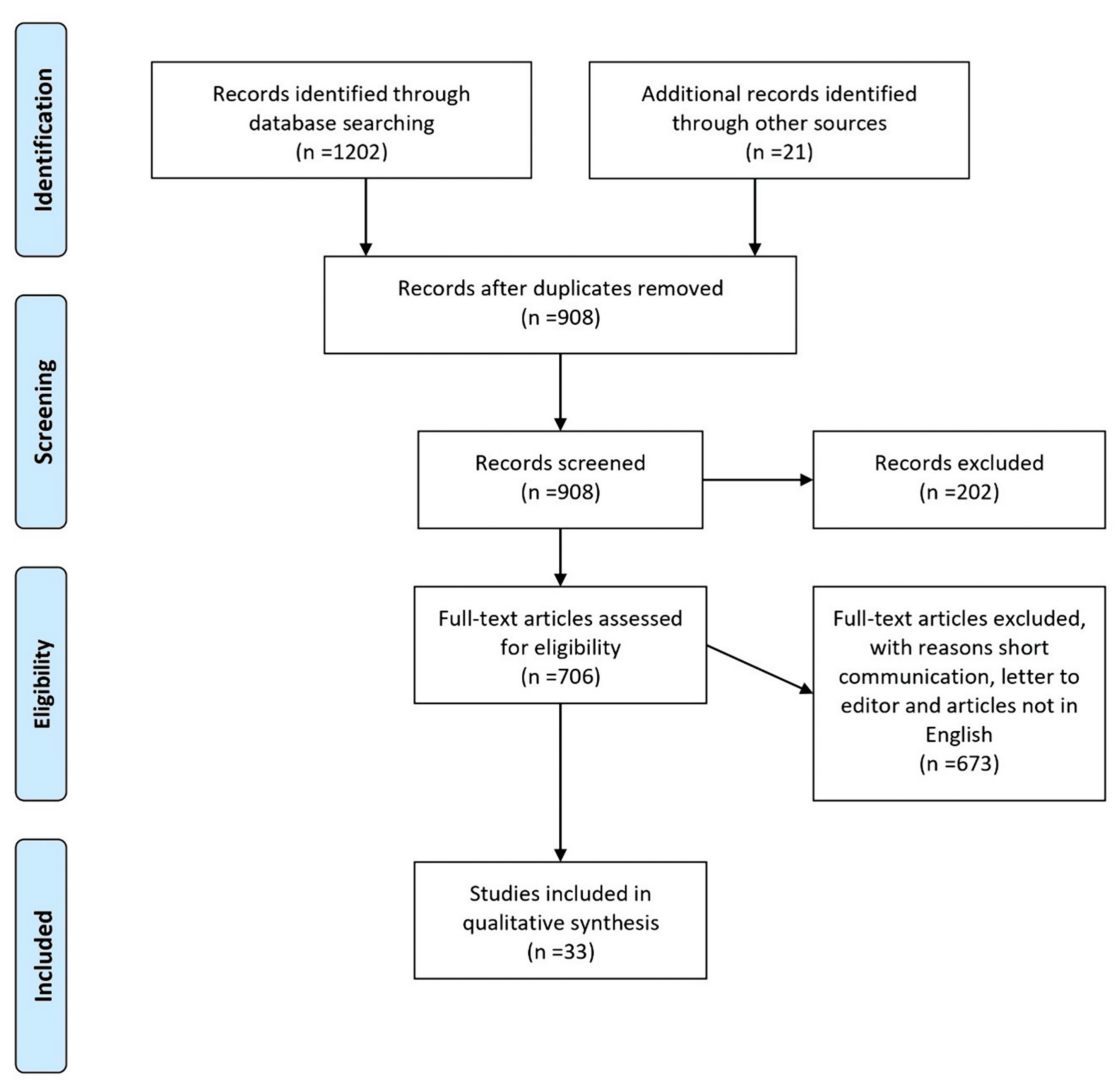
The selection process of articles used in this study. Adopted from the Preferred Reporting Items for Systematic Reviews and Meta-Analyses (PRISMA).

This review amalgamates the articles to assess various post-operative antibiotics' negative neurologic and cardiological implications. A variety of articles published on this basis are included in this article. The following databases were used to search the literature: PubMed, Scopus, Web of Science, and Google Scholar. This review includes all English-language articles and Medical Subject Headings (MeSH) terms, including “post-operative antibiotics,” "adverse drug reactions in the elderly," "neurological ADRs in the elderly," "antibiotics and neurology,” cardiac manifestations in antibiotics,” “frailty,” and “frailty index.” Articles containing systematic reviews, narrative reviews, comprehensive reviews, original research articles, case reports, and letters to the editors were included (Figure 1).

Post-operatively, treatment protocol in otorhinolaryngological surgeries mainly comprises antibiotics and analgesics to prevent infection and reduce inflammation and pain. The antibiotics most commonly prescribed to a patient can be beta-lactams and fluoroquinolones, macrolides, and so on. Third-generation cephalosporins play a significant part in post-operative antimicrobial therapy, particularly in cases of ear surgeries. These drugs are well known to have possible gastrointestinal effects and anaphylactic effects, but the consequences on the neurocognitive and cardiac functions are not that commonly discussed.

Neuropsychiatric effects of various antibiotics on the elderly

Antibiotics have side effects, which include psychiatric symptoms like mild insomnia to severe delirium and psychosis [4]. The mechanism of action varies for different classes of antibiotics. The increased odds of psychosis have been observed in various classes of drugs, such as penicillin, cephalosporins, metronidazole, fluoroquinolones, macrolides, and aminoglycosides. Among these, fluoroquinolones and metronidazole show greater incidences of psychosis [4].

Fluoroquinolones

The most commonly reported manifestations of toxicity of fluoroquinolone such as ciprofloxacin, levofloxacin, and moxifloxacin are excitatory and can range from mania, psychosis, and insomnia to confusion, hallucinations, and delusions [1]. This is due to the similarity of the structure of fluoroquinolones to the gamma-aminobutyric acid (GABA) neurotransmitter. They replace GABA from the receptor binding site due to the side chain located at the seventh position, which can also cause epileptogenic neurotoxicity [5-8]. Additionally, the N-methyl D-aspartate receptor activation is also affected by fluoroquinolones acting as GABA antagonists along with interference in GABA transmission [8,9]. Patients can be at risk of neurotoxicity when taking nonsteroidal anti-inflammatory drugs (NSAIDs) with ciprofloxacin. This is because NSAIDs can decrease renal flow and cause retention of the drugs, leading to further neurotoxicity. This interaction is due to an unsubstituted piperazinyl ring in the seventh position of the quinolone ring of ciprofloxacin [10].

Macrolides

Macrolides like erythromycin and its derivates like clarithromycin and azithromycin are most commonly used in community settings [1]. Neurological symptoms are more likely to be seen with erythromycin and clarithromycin. The GABA-A antagonistic properties in clarithromycin contribute to the direct CNS neurotoxic effect. They may cause nonconvulsive status epilepticus (NCSE) along with depressive symptoms like confusion and obtundation or excitatory symptoms like insomnia, delirium, agitation, and psychosis [11,12]. Some authors hypothesize that macrolides may inhibit cytochrome P450, which helps elevate the serum concentrations of these drugs and raises the likelihood of neurotoxic effects [11]. Meanwhile, drug interactions seen with an affinity for cytochrome P450 also have a major role in neurotoxicity.

*Beta* *Lactams*

CNS manifestations are seen in patients with advanced age, renal dysfunction, and excessive dosing. CNS toxicity from beta-lactam exposure is linked to the core ring in this class of drugs [13].

Penicillin

In 1945, Johnson and Walker were the first to describe the epileptogenic properties of penicillin [14]. The most neurotoxic events were commonly associated with piperacillin and tazobactam among the penicillin agents [1]. The subjects may experience several neurotoxic symptoms, such as convulsions, myoclonus, hallucinations, sleepiness, disorientation, odd behavior, delirium, and confusion due to piperacillin and tazobactam accumulation [15]. Predisposing conditions, like malnutrition, hypoalbuminemia, and inflammation, may increase the risk of renal dysfunction in patients, which may lead to the accumulation of antibiotics, leading to a further risk of neurotoxicity [16]. Although piperacillin and tazobactam neurotoxicity are more common in the elderly, penicillin is generally considered safe for administration.

Cephalosporins

Cephalosporins may affect the CNS with conditions like myoclonus, altered mental status, seizures, and delirium. These characteristics are present in all four generations of cephalosporins [17]. Cefazolin a first-generation cephalosporin, cefuroxime of the second generation, ceftazidime of the third, and cefepime of the fourth generation of cephalosporins are said to manifest maximum neurotoxicity [17]. The development of NSCE is said to be associated with cefepime, according to numerous reports [1,18]. Cefepime antagonizes GABA and can easily penetrate the CNS. It's used in high doses for critically ill patients, making it particularly dangerous [19].

Carbapenems

Manifestations of neurotoxicity caused by carbapenems include hallucinations, asterixis, myoclonic jerks, and cognitive impairment [1]. The basic amino acid present in the C-2 side chain of the carbapenems determines the manifestation of convulsions in the patients [20]. Meropenems and newer carbapenems have lesser neurotoxicity than imipenem and ertapenem [1,20]. Psychosis syndrome with hallucinations, including auditory and visual, and delusions have also been reported to occur due to the use of carbapenems [21].

Oxazolidinone

Linezolid is the most frequently prescribed drug among oxazolidinones in otorhinolaryngology. Neurotoxicity may manifest as peripheral neuropathy, serotonin syndrome, encephalopathy, optic neuropathy, and delirium [1]. Linezolid binds to 23S ribosomal RNA (rRNA) affecting bacterial protein synthesis and can also affect mitochondrial protein synthesis [22]. This binding to mitochondrial 16S rRNA is responsible for neurotoxicity, causing peripheral and optic neuropathy. Linezolid inhibits the metabolism of serotonin by acting as a reversible, nonselective monoamine oxidase (MAO) inhibitor, causing serotonin syndrome consisting of neuromuscular abnormalities, autonomic hyperactivity, and altered mental status [22].

Metronidazole

Metronidazole administration may cause nerve damage, leading to symptoms such as tingling, seizures, ataxic gait, slurred speech, and brain dysfunction [23]. The exact mechanism of metronidazole-induced neurotoxicity is still unclear. However, studies suggest axonal degeneration can be caused by inhibiting RNA protein synthesis by metronidazole metabolites [24]. One proposed mechanism suggests that the cerebellar and vestibular systems could be modulated by the inhibitory neurotransmitter GABA receptor [25]. Neuropsychiatric manifestations are psychosis, including mental confusion, agitation, and hallucinations, more so when combined with disulfiram as they inhibit the metabolism of dopamine-β-hydroxylase, an enzyme needed to convert dopamine to norepinephrine [26].

Aminoglycosides

Aminoglycosides, namely, gentamycin, tobramycin, neomycin, and kanamycin, are used in cases of gram-negative infections. Ototoxicity, peripheral neuropathy, encephalopathy, delirium, and neuromuscular blockade are some of the neurotoxic effects of this group of drugs [1]. Aminoglycosides can cause hearing loss by accumulating in the inner ear, generating harmful reactive oxygen species, disrupting cell signaling lipids, altering gene expression, and triggering cell death pathways [1]. Some cases have also mentioned delirium and other neurologic dysfunctions in geriatric patients undergoing treatment with tobramycin and gentamycin [1].

Adverse cardiac manifestations of antimicrobials

The major cardiac manifestations in post-operative antibiotic administration majorly include QT interval prolongation which may lead to ventricular tachycardia, torsades de pointes (TdP), and sudden cardiac arrest. However, the mechanism of action of various classes of antibiotics may or may not be different and produce similar effects when given singly or in combination.

Fluoroquinolones

According to research, some fluoroquinolone-related cardiac manifestations included prolonged QT interval by blocking cardiac rapid delayed rectifier potassium channels through interactions with their subunits' S6 aromatic amino acid residues [27,28]. The action potential duration is prolonged, which predisposes to early depolarizations and eventually leads to a vulnerability to TdP.

Macrolides

Macrolides like azithromycin, clarithromycin, and erythromycin present similar cardiac side effects as fluoroquinolones, i.e., QT prolongation leading to malignant polymorphic ventricular tachycardias, which may degenerate into ventricular fibrillation and cause sudden death [29]. The mechanism of action remains the same with an additional inhibition of cytochrome p450 enzyme, which decreases the metabolism of other QT-prolonging drugs, e.g., terfenadine [29].

Beta Lactams

Combinations of penicillin are associated with a high risk of diarrhea, which can lead to significant electrolyte abnormalities and indirectly increase the risk of TdP/QTP. However, cephalosporins, particularly ceftriaxone, have been known to cause asystole and cardiac failure in three to four cases, resulting in fatalities after an anaphylactic reaction [30].

Metronidazole

Metronidazole is an azole derivative that acts as an inhibitor of CYP3A4 and CYP2C9, resulting in QT-prolongation and its interactions with other QT prolongation agents.

Frailty and post-operative considerations

Primary frailty is an age-related issue marked by declines in various physiological systems, and it is linked to a higher risk of mortality and unplanned hospitalization [31]. It not only means age or age-related disease, but it also refers to a geriatric syndrome that includes various financial burdens, including medications, consultancies, and hospital charges [32]. To offer early help and comprehensive care in primary healthcare, it's crucial to identify elderly people with or at risk of frailty. This approach aligns well with physical and mental health models [33]. The Comprehensive Geriatric Assessment is considered the gold standard method for determining the frailty index in the geriatric population [33]. It refers to a diagnosis focused on the patient’s physical, psychological, and physiological capability to form an integrated and multidisciplinary treatment plan of treatment [2]. Another model used to determine the frailty index is the Cumulative Deficit Model. It comprises certain variables like clinical features, diseases, disabilities, and abnormalities detected in laboratory tests [34]. Initially, with 92 variables, this index can be abridged to 30 without losing the validity of the prediction [34]. In rural setups in India, the number of patients seen in senior age groups prevails in millions. Hence, it is essential to create a well-suited frailty index parameter that can be incorporated into the setting of rural hospitals. FIRE-MADE is one such frailty index devised to accommodate the rural populations of India [31]. It consists of 10 parameters which are extracted from the combination of both Comprehensive Geriatric Assessment and Cumulative Deficit Model consisting of (1) mental status, (2) activities of daily living, (3) depression, and events like (4) polypharmacy, (5) diabetes mellitus, (6) ischaemic heart disease, (7) chronic obstructive pulmonary disease/asthma, (8) stroke, (9) cancer, and (10) others [31]. Similarly, sarcopenia is another geriatric syndrome closely related to frailty and has been used as a predictor of outcomes in critically ill patients [35]. There is a need to assess the utility of sarcopenia as a predictor of post-operative antibiotic complication risk among elderly patients undergoing otorhinolaryngologic surgeries. This is especially important with an increasing number of head and neck surgeries and skull base surgeries in the elderly with the need for high post-operative antibiotic coverage. Based on the observation of various neuropsychiatric and cardiac side effects due to drugs used in the post-operative period in the elderly population, the frailty index, which has parameters related to the affection of CNS and cardiac status, as well as other effects of polypharmacy, can help in decision-making regarding the choice of post-operative antibiotics in elective surgeries especially in the field of otorhinolaryngology. The studies which are mentioned in this article are summarised in Table 1.

**Table 1:** Summary of all the studies referred in this article

Name of Author(s)	Year of Study	Key Findings
Mattappalil A, Mergenhagen KA [1]	2014	Discussed neurotoxicity associated with antimicrobial use in elderly patients.
Ellis G et al. [2]	2011	Highlighted the importance of comprehensive geriatric assessments for older adults admitted to hospitals.
Kulminski AM et al. [3]	2008	Found that cumulative deficits provide a better characterization of susceptibility to death in elderly people than phenotypic frailty.
Essali N, Miller BJ [4]	2020	Explored the potential of psychosis as an adverse effect of antibiotics.
Quigley CA, Lederman JR [5]	2004	Reported a case of a seizure possibly induced by gatifloxacin.
Asensio-Sánchez VM et al. [6]	2007	Described visual hallucinations secondary to ciprofloxacin treatment.
Isaacson SH et al. [7]	1993	Documented a case of ciprofloxacin-induced complex partial status epilepticus.
Annadatha A et al. [8]	2019	Reported a case of levofloxacin-induced psychosis in an elderly patient.
Schmuck G et al. [9]	1998	Studied the excitatory potencies of fluoroquinolones in the central nervous system using an in vitro model.
Desai C [10]	2016	Provided insights from Meyler's encyclopedia on adverse drug reactions and interactions.
Bandettini di Poggio M et al. [11]	2011	Investigated clarithromycin-induced neurotoxicity in adults.
Zareifopoulos N, Panayiotakopoulos G [12]	2017	Reviewed the neuropsychiatric effects of antimicrobial agents.
Chow KM et al. [13]	2005	Discussed beta-lactam antibiotics' neurotoxicity from bench to bedside.
Walker AE et al. [14]	1946	Described the convulsive effects of antibiotic agents on the cerebral cortex.
Raichle ME et al. [15]	1971	Examined the neurotoxicity of intravenously administered Penicillin G.
Huang W-T et al. [16]	2009	Reported a case of neurotoxicity associated with standard doses of piperacillin in an elderly patient with renal failure.
Grill MF, Maganti R [17]	2008	Discussed cephalosporin-induced neurotoxicity, including clinical manifestations and potential pathogenic mechanisms.
Yahav D et al. [18]	2007	Conducted a systematic review and meta-analysis on the efficacy and safety of cefepime.
Payne LE et al. [19]	2017	Provided a systematic review on cefepime-induced neurotoxicity.
Sunagawa M et al. [20]	1995	Investigated structural features resulting in the convulsive activity of carbapenem compounds.
Oo Y et al. [21]	2014	Reported a case of psychosis and encephalopathy associated with ertapenem use.
Narita M et al. [22]	2007	Discussed the association of linezolid with peripheral and optic neuropathy, lactic acidosis, and serotonin syndrome.
Alston TA [23]	1985	Provided early insights into the neurotoxicity of metronidazole.
Caylor KB, Cassimatis MK [24]	2001	Reported metronidazole neurotoxicosis in cats, providing relevant analogical insights for human cases.
Kuriyama A et al. [25]	2011	Conducted a systematic review on metronidazole-induced central nervous system toxicity.
Luykx JJ et al. [26]	2013	Reported psychotic symptoms after combined use of metronidazole and disulfiram.
Anderson ME et al. [27]	2001	Explored the potassium current antagonist properties and proarrhythmic consequences of quinolone antibiotics.
Alexandrou AJ et al. [28]	2006	Investigated the mechanism of hERG K+ channel blockade by moxifloxacin, a fluoroquinolone antibiotic.
Guo D et al. [29]	2010	Conducted a systematic review on the cardiotoxicity of macrolides.
Saritas A et al. [30]	2012	Reported asystole after the first dose of ceftriaxone, highlighting its potential for causing severe cardiac side effects.
Kumar S et al. [31]	2019	Developed and validated a modified Frailty Risk Index as a predictor of mortality in rural elderly populations.
Ahmed N et al. [32]	2007	Discussed frailty as an emerging geriatric syndrome.
Turner G, Clegg A [33]	2014	Provided best practice guidelines for the management of frailty, developed by the British Geriatrics Society, Age UK, and Royal College of General Practitioners.
Clegg A et al. [34]	2016	Developed and validated an electronic frailty index using routine primary care electronic health record data.
Bhurchandi S et al. [35]	2021	Investigated the correlation of sarcopenia with a modified frailty index as a predictor of outcomes in critically ill elderly patients.

Limitations

The article focuses solely on the neuropsychiatric and cardiac effects of antibiotics and frailty assessment. It does not cover the interactions of multiple antibiotics or their combined effects. The study only looks at the individual properties of antibiotics, not their combinations. The use of post-operative antibiotics in ear, nose, and throat surgeries is also changing with newer antibiotics. However, this study only discusses commonly used antibiotics excluding the antifungal and anti-tubercular drugs. While there are numerous studies on frailty indices for the elderly, only a few are highlighted in this particular study.

## Conclusions

A pre-operative assessment in elderly patients should include a frailty assessment risk regarding the cognitive component, which would not only help in identifying the risk of post-operative medications but also the capability to handle overall post-surgical stress. In patients with higher frailty index, the drugs likely to cause neuropsychiatric and cardiac side effects should be avoided either alone or in combination to reduce the incidences of post-surgical complications which may lead to morbidity and mortality.
